# Virtual Reality–Based Avatar Intervention for Eating Disorders: Mixed Methods Feasibility Study

**DOI:** 10.2196/88445

**Published:** 2026-03-24

**Authors:** Nina Kappel Hansen, Emma Slebsager Ries, Katrine Rasmussen, Valentina Cardi, Anne Bryde, Carsten Hjorthøj, Nadia Micali, Thomas Ward, Louise Birkedal Glenthøj

**Affiliations:** 1 VIRTU Research Group Mental Health Center Copenhagen Copenhagen University Hospital Hellerup, Capital Region Denmark; 2 Department of Psychology University of Copenhagen Copenhagen, Capital Region Denmark; 3 Department of General Psychology University of Padova Padova Italy; 4 Center for Eating and feeding Disorders Research Mental Health Center Ballerup Copenhagen University Hospital Ballerup, Capital Region Denmark; 5 Mental Health Center Copenhagen Copenhagen University Hospital Hellerup, Capital Region Denmark; 6 Section of Epidemiology Department of Public Health University of Copenhagen Copenhagen Denmark; 7 Institute of Psychiatry Psychology & Neuroscience King’s College London London United Kingdom; 8 South London & Maudsley NHS Foundation Trust London United Kingdom

**Keywords:** Virtual reality-assisted therapy, eating disorders, anorexia nervosa, bulimia nervosa, eating disorder voice, anorexic voice, externalization techniques, narrative therapy, relational therapy

## Abstract

**Background:**

There is a growing interest in developing novel psychological interventions for eating disorders, with an emphasis on targeting maintaining factors. One hypothesized mechanism underlying illness maintenance is the experience of an “inner eating disorder voice,” which reinforces maladaptive thoughts, emotions, and behaviors. Preliminary studies suggest that the eating disorder voice is common among patients and is linked to greater illness severity.

**Objective:**

This single-arm, mixed methods pilot feasibility study aimed to evaluate a novel virtual reality (VR)–based therapy targeting the eating disorder voice. The intervention was adapted from AVATAR therapy for psychosis and was examined as an adjunct to treatment as usual in individuals with eating disorders. In this adaptation, participants engaged with a therapist-controlled avatar representing their inner eating disorder voice in VR. The primary objectives were to assess the feasibility, acceptability, and safety of the intervention and to provide preliminary estimates of its clinical efficacy.

**Methods:**

Adults with anorexia nervosa (9/10, 90%) or bulimia nervosa (1/10, 10%) took part in a 7-session VR-based therapy course at the Mental Health Centre Copenhagen, Copenhagen University Hospital, Denmark, alongside their treatment as usual. Quantitative measures of feasibility (recruitment, retention rates, and satisfaction scores), safety, and eating disorder–related outcomes were collected at baseline and after treatment between June 2023 and January 2024. Qualitative interviews conducted after the intervention (October 2023 to November 2023) explored participants’ experiences. Descriptive statistics, paired *t* tests, and thematic analysis were conducted, and the analyses were finalized in October 2025.

**Results:**

Recruitment targets were met: 14 individuals were referred, and 11 provided consent well within the prespecified time frame. Treatment completion rate was 80% (8/10; 95% CI 44%-97%), and no serious adverse events occurred. Participants reported high satisfaction (7/10, 70%; mean 9, SD 1.15 on a 10-point Likert scale; median 9, IQR 8.5-10.0; range 7-10), and qualitative data (8/10, 80%) suggested that they valued the immersive virtual representation of their eating disorder voice. Exploratory analyses indicated improvements in eating disorder symptoms (Hedges *g*=−0.99, 95% CI −1.74 to −0.24; *P*=.01), power dynamics associated with the eating disorder voice (Hedges *g*=−1.63, 95% CI −2.59 to −0.67; *P*=.002), and emotion regulation via cognitive reappraisal (Hedges *g*=0.87, 95% CI 0.08-1.66; *P*=.04).

**Conclusions:**

The VR-based avatar intervention for eating disorders was feasible, acceptable, and safe, with preliminary signals of clinical improvement. These findings support further development and evaluation of the intervention in a randomized clinical trial.

## Introduction

Eating disorders involve harmful eating behaviors and an excessive focus on weight and body shape in one’s self-evaluation [1]. These illnesses impact at least 7% of the global population [2] and impose severe physical and psychological consequences, including an elevated risk of early mortality [3-5]. Additionally, eating disorders impose substantial economic burdens, including health care expenditures, reduced employment rates, and productivity losses, with overall costs exceeding those associated with anxiety and depression [6].

Psychological therapies are recommended as first-line treatments for eating disorders [7], although their effectiveness, particularly for anorexia nervosa, remains suboptimal [8-10]. Approximately 30% of adult patients who complete treatment relapse within a year of treatment completion [11,12], and dropout rates ranging from 20% to 70% have been reported [13,14]. Long-term recovery rates remain low, especially for anorexia nervosa [15], while barriers to initiating treatment remain high [16,17]. These challenges underscore the need for innovative and engaging therapeutic approaches that target core maintaining mechanisms of eating disorder psychopathology and enhance treatment engagement.

An estimated 94% of individuals with an eating disorder report experiencing an internal “eating disorder voice,” often described as an inner self-critical commentary or self-talk focused on weight, eating, and self-worth [18,19]. While this voice may be perceived as supportive at first, it typically becomes increasingly critical and controlling, reinforcing harmful eating behaviors [19]. Qualitative research indicates that this shift can feel like a division between the disorder and the self, with the eating disorder voice becoming a dominant, dissociated part of the self that often overrides personal values and leaves individuals feeling powerless [20,21].

A stronger eating disorder voice is associated with more frequent compensatory behaviors (eg, fasting, vomiting, laxative misuse, and compensatory exercise) and longer illness duration [22]. Accordingly, research suggests that an individual’s subordinate stance toward the eating disorder voice may play a significant role in maintaining eating disorder symptoms and hindering treatment engagement [22]. Despite its clinical significance, the everyday experience of the eating disorder voice and the individual’s dialogue with it are rarely addressed directly in therapy. Most treatment programs aim to reduce the disorder’s dominance over identity and behavior; however, specifically targeting the eating disorder voice in a relational manner may further empower patients to reclaim agency and develop a self-concept not defined by the disorder [19,21,23,24].

Similar relational dynamics are observed in individuals with psychosis who hear distressing voices, inspiring adaptations of relational approaches such as “voice dialogue” for eating disorders. Preliminary qualitative work has used chair work techniques to facilitate dialogue between patients and their eating disorder voice [21,24].

AVATAR therapy, a digital intervention initially developed for psychosis, uses computer-generated avatars to embody distressing voices and enable structured dialogue. It has demonstrated efficacy in reducing voice severity in 2 fully powered randomized clinical trials [25-27]. A proof-of-concept study and a recent feasibility study adapting AVATAR therapy for anorexia nervosa suggest that this approach is feasible, acceptable, and potentially effective for shifting power dynamics, enhancing self-compassion, and reducing symptoms of stress and anxiety in individuals with anorexia nervosa [14,23].

The previous adaptation of AVATAR therapy for anorexia nervosa was delivered on a standard desktop screen [14,23]. This study is the first to evaluate a virtual reality (VR)–based format, providing a fully immersive environment that may enhance therapeutic engagement through increased presence and exposure to emotionally salient content [28]. Although most studies on the eating disorder voice focus on anorexia nervosa, qualitative findings suggest that individuals with bulimia nervosa report similar inner voices [29]. Additionally, to test the intervention more broadly, the target population was extended to include patients with bulimia nervosa. The primary aim was to assess the feasibility, acceptability, and safety of this novel VR-based avatar intervention while collecting exploratory data on its potential clinical effects. Qualitative interviews were incorporated to capture participant experiences, refine the intervention, and inform the design of a future randomized clinical trial.

## Methods

### Trial Design

The study was a single-arm pilot feasibility trial enrolling 10 participants with anorexia nervosa or bulimia nervosa. All participants received the VR-based avatar intervention alongside their ongoing standard treatment, which consisted of long-term weekly psychodynamic therapy sessions with a psychologist and, for some, additional dietary guidance, focused on symptom management rather than full recovery. The research team conducted assessments of eating disorder–related outcomes and quality of life at baseline and after the treatment (12 weeks after baseline) and completed semistructured qualitative interviews after the intervention. The therapist monitored safety throughout the study. The therapy sessions, assessments, and qualitative interviews were conducted at the Mental Health Centre Copenhagen, Copenhagen University Hospital, Denmark, between June 2023 and January 2024. The study is reported in accordance with the CONSORT (Consolidated Standards of Reporting Trials) extension for pilot and feasibility trials, the TIDieR (Template for Intervention Description and Replication) guidelines, and the SRQR (Standards for Reporting Qualitative Research). Completed checklists are provided in Multimedia Appendices 1-3.

### Ethical Considerations

The study was approved by the Committee on Health Research Ethics of the Capital Region of Denmark (H-22067692) and the Danish Data Agency (P-2022-932). Participants received oral and written information about the study and provided written informed consent before enrollment. Study data were deidentified before analysis and stored on secure, password-protected servers in accordance with the requirements of the Danish Data Agency. No compensation was provided to participants in accordance with Danish legislation restricting financial or material compensation for patient participation.

### Recruitment

Participants were recruited from a specialist service within the Mental Health Services of Copenhagen, which provides a multidisciplinary treatment program including individual or group psychotherapy, dietetic support, and social work services for patients with long-standing eating disorders. Between May 2023 and October 2023, clinical staff from the specialist service identified potential participants who met the inclusion criteria and approached them with study information. All study materials were reviewed by a patient with lived experience and revised accordingly. With participants’ consent, clinicians forwarded their contact information to the research group via a secure email system. Members of the research team then contacted potential participants by telephone to assess eligibility. Individuals who met the inclusion criteria and expressed interest were invited to a baseline interview, during which written informed consent was obtained.

Inclusion criteria were (1) age of 18 years or above, (2) an ICD-10 diagnosis of anorexia nervosa or bulimia nervosa (ICD-10 codes F50.0, F50.1, F50.2, and F50.3) [30], (3) self-reported recognition of an internal eating disorder voice, and (4) proficiency in Danish or English. Individuals with a diagnosis of psychosis and/or those at risk of suicidal behavior were excluded. Eligibility criteria remained unchanged after trial commencement.

### Sample Size

The study involved 10 participants, consistent with pilot and feasibility guidance prioritizing procedures, refinement, and parameter estimation over efficacy [31]. This sample size was adequate to assess recruitment and retention, acceptability, and safety and to generate preliminary data for a larger randomized controlled trial.

### Intervention

This study adapted a previously validated 3D VR-based avatar intervention for psychosis (the “Challenge” trial) [26] to target eating disorder psychopathology. The adaptation involved collaboration with 8 individuals with lived experience of eating disorders, whose input informed the VR content and the therapy manual to ensure clinical relevance and acceptability prior to commencement of the pilot study.

Participants received 7 weekly individual VR-based avatar sessions alongside their ongoing standard treatment. The VR-based avatar intervention was delivered by a psychologist with clinical experience in the treatment of eating disorders and formal training in avatar-based therapy acquired through our previous psychosis trial. All VR-based avatar therapy sessions were conducted face-to-face in a dedicated therapy room.

In the initial session, participants worked with the therapist to identify key distressing verbal content and characteristics of the eating disorder voice. They then designed a computer-generated avatar to embody their voice, with options to customize sex, age, facial features, body size, skin color, and hair color. A voice modulation program was used to adjust the therapist’s voice to match the tone and pitch of the participant’s imagined eating disorder voice.

In subsequent sessions, participants wore a VR headset and noise-canceling headphones while engaging in dialogue with the avatar. Each session lasted 60 minutes, including approximately 15 minutes spent interacting in VR with the avatar, with the remainder dedicated to preparation and reflection.

**Figure 1:**
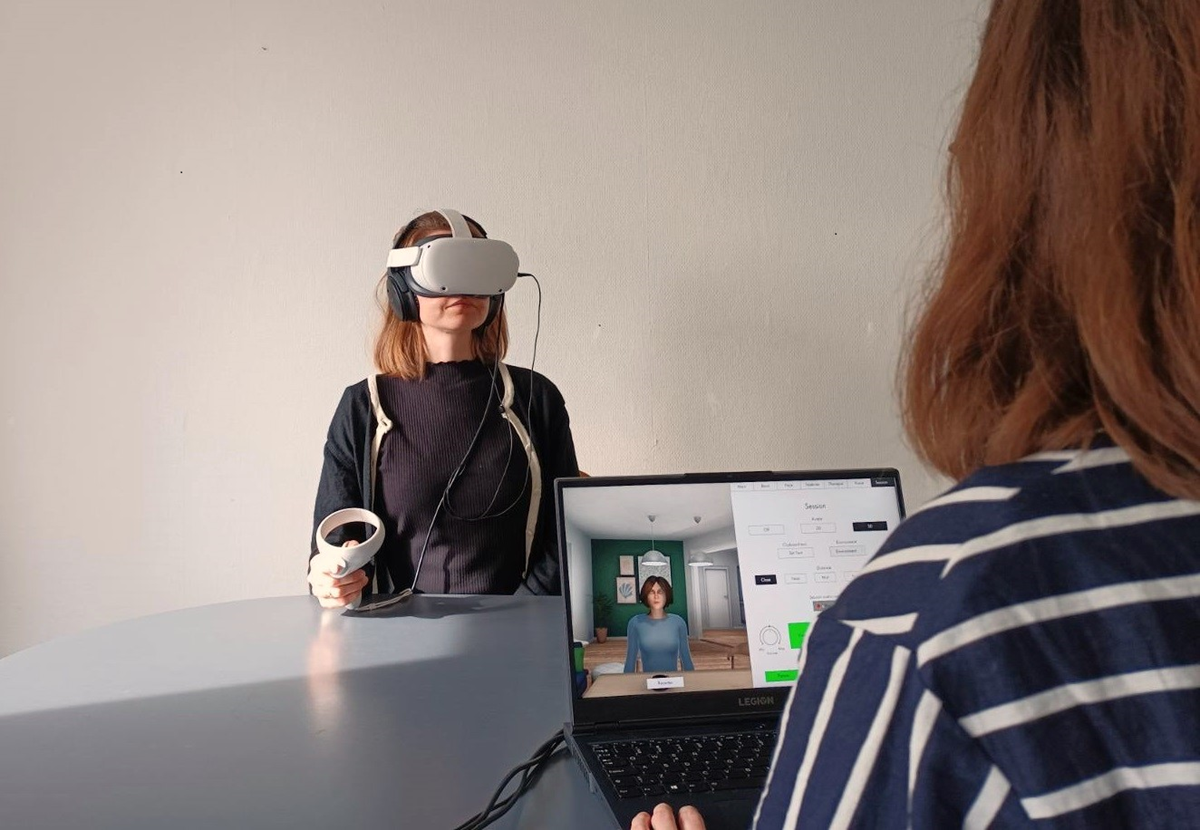
Photograph of the therapy setup and virtual reality (VR) equipment used in a single-arm feasibility study of VR-based avatar therapy for eating disorders conducted at the Mental Health Centre Copenhagen, Copenhagen University Hospital, Denmark, between June 2023 and January 2024.

In the virtual environment, participants were placed in a living room setting facing the avatar. The therapist was not visible in VR but was present in the therapy room, controlling the avatar and facilitating the interaction by alternating between speaking as the avatar and as a supportive therapist (Figure 1). When speaking as the avatar, the therapist used a transformed voice, and the avatar’s mouth movements were synchronized with the speech to represent the participant’s eating disorder voice.

Participants were encouraged to confront the avatar and assert their beliefs, values, and wishes, with the aim of gaining increased control over their eating disorder voice. Early sessions followed a structured format with prescripted dialogue, gradually progressing to more spontaneous exchanges to increase immersion. The therapist continuously monitored the participants’ distress levels and adjusted the intensity of interactions as needed.

To promote skill consolidation, participants were provided with a picture of their avatar and audio recordings of avatar dialogues for between-session review. The recordings aimed to reinforce participants’ sense of control and motivation for change; maintain therapeutic gains; and support discussions with family members, peers, or other treatment providers.

### Outcome Measures

#### Primary Outcomes: Feasibility, Acceptability, and Safety

Primary outcomes included feasibility and acceptability of the therapy, operationalized as recruitment of 80% or more of the target sample (N=10) in 12 months, 80% or higher retention to the study protocol at cessation of therapy (7 sessions), and 80% (8/10) or more of the participants reporting a satisfaction rating of 7 or higher on a Likert scale from 1 to 10. Acceptability was further assessed using the Client Satisfaction Questionnaire (CSQ) [32].

The Negative Effects Questionnaire was applied as a participant-reported, exploratory measure of negative effects, including a broad scope of psychosocial, emotional, and cognitive effects attributed to the treatment [33].

Safety was monitored by the therapist at each session through systematic recording of adverse events related or unrelated to participation in the study (eg, death, suicide attempts, hospital admissions, and complaints about the therapy). Any adverse events were reported to the Danish Health Research Ethics Committee, which oversaw the study and had the authority to recommend pausing or terminating the study.

#### Exploratory Clinical Outcomes

Given the novelty of the intervention, we adopted an exploratory approach to outcome measures to identify potential areas of therapeutic impact. Preliminary indications of efficacy were assessed using the following measures obtained at baseline and at the end of treatment (12 weeks after baseline): (1) Eating Disorder Examination Questionnaire (EDE-Q) global score [34] to assess eating disorder symptoms, (2) Modified Psychotic Symptoms Rating Scale–Auditory Hallucinations subscale total score [35] to assess eating disorder voice characteristics and the distress it evokes, (3) Body Shape Questionnaire–34 total score [36] to assess body dissatisfaction, (4) Eating Disorder Quality of Life Scale (EDQLS) total score [37] to assess quality of life, (5) Self-Compassion Scale (SCS) total score [38] to assess self-compassion, (6) Revised Beliefs About Voices Questionnaire subscale scores (for omnipotence, malevolent beliefs, affective engagement, benevolent beliefs, behavioral engagement, affective resistance, and behavioral resistance) [39] to assess engagement with the eating disorder voice, (7) Voices Power Differential Scale total score [40] to assess voice power, (8) Voices Acceptance and Action Scale (VAAS) total acceptance score [41] to assess acceptance and action toward the eating disorder voice, (9) Hospital Anxiety and Depression Scale total scores [42] to assess anxiety and depression symptoms, (10) General Self-Efficacy Scale total score to assess self-efficacy [43], (11) Emotion Regulation Questionnaire subscale scores for Cognitive Reappraisal and Expressive Suppression [44] to assess emotion regulation, (12) Identity and Eating Disorder Questionnaire (IDEA) total score [45] to assess identification with the eating disorder and sense of embodiment, and (13) Behavior Rating Inventory of Executive Function global score [46] to assess executive functioning.

### Statistical Analysis

Feasibility, acceptability, and safety outcomes were summarized descriptively [31,47]. Recruitment, retention, and follow-up rates were reported as proportions with Clopper-Pearson 95% CIs. Acceptability was assessed with patient satisfaction scores (Likert scale 1-10), with a mean score of 7 or higher reported by 80% (8/10) of more of the participants indicating acceptability. Safety was described using frequencies of adverse events. Exploratory efficacy analyses used 2-tailed paired *t* tests (α=.05) comparing baseline and posttreatment scores. For each measure, mean differences, SDs, medians, IQRs, *P* values, and Hedges g with CIs were stated. Due to a REDCap (Research Electronic Data Capture) error, 1 item from the IDEA questionnaire and the first 12 items of the EDQLS were missing at baseline for 9 participants. To ensure comparability across time points, analyses excluded these items from both baseline and follow-up calculations. No data were excluded.

### Qualitative Analysis

Semistructured interviews were conducted to explore participants’ experiences regarding barriers and facilitators to engagement, with the aim of informing intervention acceptability and refinement [31,47]. A total of 8 interviews were conducted by 2 members of the research group between October 2023 and November 2023. The interviews followed a flexible guide based on the study aims but allowed participants to highlight their own perspectives. Data were analyzed thematically using the framework by Braun and Clarke [48], with a hybrid deductive-inductive approach: predefined codes reflected study objectives, while additional codes and themes emerged inductively from the data. To enhance rigor, the 2 researchers independently coded the transcripts, discussed discrepancies, and agreed on the final coding structure. To address potential bias, given that interviewers were members of the research team, reflexivity practices were applied throughout. NVivo (version 15.3.1; Lumivero) software was used to support coding and data management.

## Results

### Participant Characteristics

**Figure 2:**
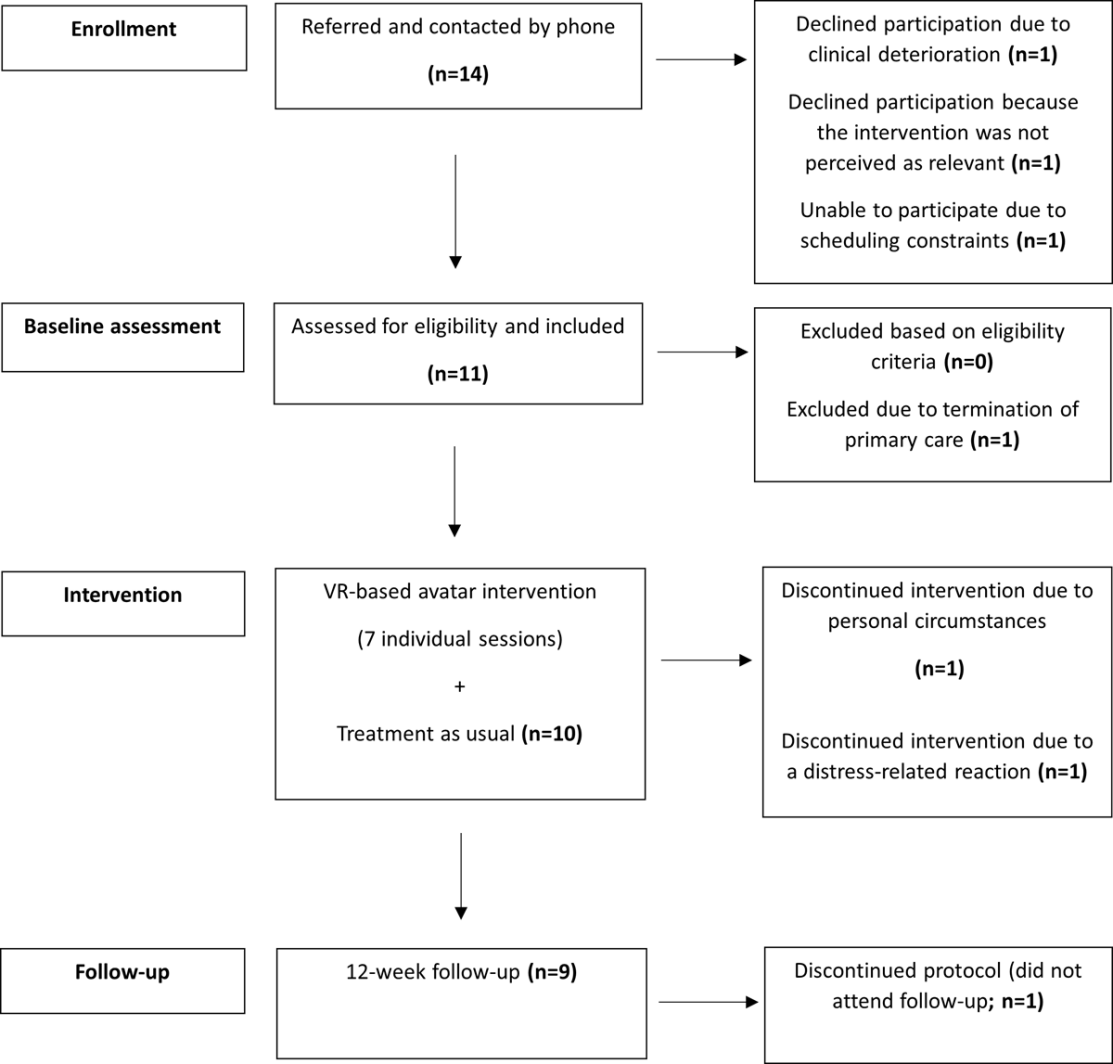
CONSORT (Consolidated Standards of Reporting Trials) flow diagram of participant (N=10) enrollment, intervention, and follow-up in a single-arm feasibility study of a virtual reality (VR)–based avatar therapy for eating disorders conducted at the Mental Health Centre Copenhagen, Copenhagen University Hospital, Denmark, between June 2023 and January 2024.

A total of 14 eligible patients were referred, of whom 3 (21%) declined participation. In total, 79% (11/14; 95% CI 49%-95%) consented and completed the baseline assessment. Another participant (1/14, 7%) was excluded after baseline due to discontinuation of standard care. Consequently, 71% (10/14) of the participants referred received the intervention as planned (Figure 2). After study commencement, 10% (1/10) of the participants were identified as having nonorganic psychosis, characterized by a single persistent auditory hallucination without other psychotic features. Given the symptom profile, the participant remained in the study, and the data were included in the analyses.

A total of 10 participants (all female) were enrolled in the study, with a median age of 35.7 (IQR 26.2-40.8; range 23.0-55.0; mean 35.1, SD 9.8) years. Of the 10 participants, 1 (10%) had bulimia nervosa, whereas the remaining 9 (90%) had anorexia nervosa. The median illness duration was 16.5 (IQR 11.3-19.8; range 7.0-45.0; mean 17.8, SD 10.7) years. Eating disorder severity, measured using the EDE-Q global score, averaged 3.44 (SD 1.56; median 3.9, IQR 2.9-4.1; range 0.3-5.2), reflecting symptoms in the mild to moderate range. The mean BMI was 19.4 (SD 1.6; median 19.1, IQR 18.7-20.2; range 16.6-21.7) kg/m^2^. A total of 90% (n=9) of the participants reported psychiatric comorbidity (ie, generalized anxiety, nonorganic psychosis, autism spectrum disorder, posttraumatic stress disorder, panic attacks, attention-deficit/hyperactivity disorder, personality disorder, bipolar disorder, and obsessive-compulsive disorder) and all were receiving psychiatric medication (Table 1).

**Table 1:** Baseline characteristics of participants enrolled in a single-arm feasibility study of virtual reality–based avatar therapy for eating disorders conducted at the Mental Health Centre Copenhagen, Copenhagen University Hospital, Denmark, between June 2023 and January 2024 (N=10).

Characteristics		Value
**Age (y)**		
	Mean (SD)	35.1 (9.8)
	Range	23.0-55.0
	Median (IQR)	35.7 (26.2-40.8)
**Sex, n (%)**		
	Female	10 (100)
**Diagnosis of eating disorder, n (%)**		
	Anorexia nervosa	9 (90)
	Bulimia nervosa	1 (10)
**Illness duration (y)**		
	Mean (SD)	17.8 (10.7)
	Range	7.0-45.0
	Median (IQR)	16.5 (11.3-19.8)
**Eating disorder severity (** **Eating Disorder Examination Questionnaire** **total score)**		
	Mean (SD)	3.4 (1.6)
	Range	0.3-5.2
	Median (IQR)	3.9 (2.9-4.1)
**BMI (kg/m^2^)**		
	Mean (SD)	19.4 (1.6)
	Range	16.6-21.7
	Median (IQR)	19.1 (18.7-20.2)
**Comorbidity, n (%)**		9 (90)
	Generalized anxiety	1 (10)
	Nonorganic psychosis and other	1 (10)
	Phobic anxiety	1 (10)
	Posttraumatic stress disorder	3 (30)
	Attention-deficit/hyperactivity disorder	2 (20)
	Bipolar mood disorder	1 (10)
	Obsessive-compulsive disorder	1 (10)
	Autism spectrum disorder	1 (10)
	Personality disorder	1 (10)
**Psychotropic medication, n (%)**		9 (90)
	Antidepressants	6 (60)
	Antipsychotics	4 (40)
	Mood stabilizers	1 (10)
	Benzodiazepines	2 (20)

### Evaluation Outcomes

#### Feasibility

Recruitment of the target sample was completed in 5.5 months, within the prespecified 12-month time frame. Of the 10 participants, 8 (80%; 95% CI 44%-97%) completed all 7 therapy sessions. In total, 10% (1/10) of the participants withdrew after 2 sessions due to emotional distress triggered by the avatar dialogues in the context of previous trauma, and 10% (1/10) of the participants discontinued participation after 3 sessions due to personal stressors. The end-of-intervention assessment was completed by 90% (9/10; 95% CI 55%-99.7%) of the participants, although 22% (2/9) provided partial data, leaving a total of 70% (7/10) of the participants who completed the full assessment battery (Multimedia Appendix 4 shows an overview of the participants per outcome measure). In total, 80% (8/10) of the participants took part in qualitative interviews, including 10% (1/10) who had discontinued therapy early due to emotional distress.

#### Safety

No serious adverse events occurred during the intervention period (eg, deaths, suicide attempts, serious violent incidents, formal complaints, crisis referrals, or hospital admissions). One nonserious adverse event was recorded: emotional distress leading to early withdrawal.

#### Acceptability: Quantitative Analysis

The overall satisfaction rate was high, with a mean of 9 out of 10 (SD 1.15; median 9.0, IQR 8.5-10.0; range 7.0-10.0; 7/10, 70%) on a 10-point Likert scale. This aligned with a high mean score of 30.7 out of 32.0 (SD 3.5; median 28, IQR 25-29; range 22.0-30.7; 7/10, 70%) on the CSQ, measuring client satisfaction. However, responses were missing from the 2 participants who terminated therapy prematurely and from 1 participant who only partially completed the end-of-treatment assessment and did not respond to the satisfaction scale or the CSQ.

On broader self-reported negative effects, participants reported an average of 5.3 (SD 6.0; range 0-15) negative effects on the Negative Effects Questionnaire, most commonly distress-related experiences (eg, stress and sadness) and concerns about treatment quality (eg, misunderstanding of treatment and unmet expectations). The mean perceived severity was 1.2 (SD 0.9; range 0-2.2) on a 0 to 4 scale, indicating a mild overall impact.

#### Preliminary Efficacy

Regarding exploratory clinical outcomes, participants showed improvements in eating disorder symptoms and key psychological processes related to the eating disorder. The EDE-Q global score demonstrated a reduction in disordered eating behaviors (Hedges *g*=−0.99, 95% CI −1.74 to −0.24; median preintervention score=3.98, IQR 2.98-4.13; median postintervention score=2.89, IQR 2.51-3.46; *P*=.01). The Voice Power Differential Scale indicated a substantial shift in the perceived power balance between the participant and the eating disorder voice (Hedges *g*=−1.63, 95% CI −2.59 to −0.67; median preintervention score=23.0, IQR 17.75-25.75 median postintervention score=18.0, IQR 15.00-19.00; *P*=.002). Emotional engagement with the eating disorder voice, measured by the Revised Beliefs about Voices Questionnaire engagement affective subscale, decreased significantly (Hedges *g*=−0.98, 95% CI −1.80 to −0.16; median preintervention score=5.5, IQR 4.25-7.50; median postintervention score=3.0, IQR 1.00-5.50; *P*=.02). Cognitive reappraisal (eg, the ability to think differently to alter emotional states), as measured by the Emotion Regulation Questionnaire, also improved significantly (Hedges *g*=0.87, 95% CI 0.08-1.66; median preintervention score=4.1, IQR 2.67-4.50; median postintervention score=4.5, IQR 4.33-5.42; *P*=.04).

In contrast, 3 Self-Compassion Scale subscales—self-judgment (*P*<.001), isolation (*P*=.03), and overidentification (*P*<.001)—showed significant negative change, whereas 3 Self-Compassion Scale subscales—self-kindness, common humanity, and mindfulness—showed positive, nonsignificant change.

Acceptance of the eating disorder voice (measured using VAAS) decreased slightly but not significantly (*P*=.12). No significant change was observed in disease-specific quality of life (*P*=.76). Multimedia Appendix 4 displays all exploratory outcome findings.

#### Acceptability: Qualitative Analysis

##### Overview

Four main themes captured participants’ experiences of the VR-based avatar intervention. The themes reflected both the intensity of confronting the eating disorder voice and the potential therapeutic value of that intensity. Further qualitative findings will be reported in a separate paper currently in preparation.

##### Theme 1: Real-Time Visual and Auditory Externalization–Enabled Resistance

Participants described how the avatar dialogue helped externalize the eating disorder voice, making it feel more concrete, as 1 participant noted:

It’s not just thoughts flying around in my head.... It’s something that’s really being said to me.

Participant 1

This shift in perception facilitated resistance:

If it’s this avatar saying to me, “You look fat,” then it’s easier to say, “You’re not going to talk to my body like that.”

Participant 1

In general, the participants stressed that the avatar created psychological distance from the eating disorder thoughts, making it easier to challenge them.

##### Theme 2: Empowerment

Across participants, the avatar dialogue fostered a sense of empowerment, described in three ways: (1) increased control over eating disorder thoughts and behaviors, (2) enhanced motivation for change, and (3) new insights and greater self-acceptance. One participant explained the following:

It felt like being in dialogue with something I previously wasn’t able to control.

Participant 6

These subjective accounts aligned with quantitative findings of increased perceived power and reduced eating disorder symptoms.

##### Theme 3: Intensity as Catalyst and Challenge

The heightened realism of thought content intensified emotions, sometimes experienced as overwhelming or anxiety-provoking, yet was generally regarded as therapeutically meaningful:

If it affected me as much as it did, then it must have an effect.

Participant 6

For manual refinement, some participants suggested preparatory psychoeducation and postsession cooldown periods to better manage the emotional load. The participant with a trauma history highlighted the need for tailored pacing and support.

##### Theme 4: Barriers and Suggested Improvements

Technical issues (eg, sound disruptions and heavy VR goggles) and limited avatar customization reduced immersion. Several participants also recommended extending the number of sessions, noting that initial meetings were often spent acclimating, while later sessions felt more impactful and natural.

## Discussion

### Principal Findings

This mixed methods pilot feasibility study explored a novel VR-based avatar intervention used alongside treatment as usual for individuals with anorexia nervosa and bulimia nervosa. The intervention proved feasible, with a 100% recruitment rate achieved in less than 6 months, well ahead of the 12-month target, and 80% treatment adherence, meeting predefined feasibility criteria. Acceptability was high, although incomplete response rates introduce some uncertainty. Safety monitoring identified no serious adverse events; however, 1 participant discontinued due to distress triggered by the avatar dialogue, highlighting the need for individualized pacing. Participants reported a modest number of negative therapy-related events, indicating that negative experiences were present but not highly frequent across the sample. Events were generally rated as mild and most often involved distress (eg, experiencing stress or sadness from therapy). However, 1 clinician reported symptom deterioration in a patient 7 months after discontinuing treatment, for which a potential association with the intervention could not be ruled out.

The coexistence of high satisfaction and mild to moderate negative effects suggests that participants valued the intervention overall, despite experiencing some transient distress. Qualitative findings supported this interpretation, describing the avatar dialogue as meaningful and immersive, helping participants externalize and concretize the eating disorder voice, thereby making it easier to foster resistance and regain a sense of control. At the same time, the realism and intensity of the dialogues could be overwhelming, particularly early in treatment. This duality underscores the importance of preparatory psychoeducation, postsession processing, and tailored pacing. Careful assessment of potential triggers and a slower pace of exposure might be of particular importance for patients with trauma histories. Participants also recommended improving technical reliability, enhancing avatar customization, and extending the number of sessions to maximize therapeutic benefit, consistent with findings from previous feasibility research on computer-based AVATAR therapy for anorexia nervosa [23]. Further research into avatar customization options is warranted, as enhancing personalization may be important for improving therapeutic engagement in future interventions.

Despite the small sample, exploratory analyses suggested potential for clinical change across several domains, including large effect sizes for perceived power shifts in the eating disorder voice and reduced emotional engagement with the voice. In contrast to the previous AVATAR feasibility study [14], participants also reported reductions in disordered eating behaviors, and cognitive reappraisal abilities improved significantly, indicating enhanced flexibility in managing emotional responses. A nonsignificant decline in “voice acceptance” was also observed, which may represent a therapeutic shift toward greater psychological distance from the eating disorder voice.

Unexpectedly, 3 self-compassion subscales (self-judgment, isolation, and overidentification) worsened, whereas self-kindness, common humanity, and mindfulness showed nonsignificant gains. Follow-up interviews with participants showing the largest negative changes suggested that the intervention increased awareness of self-critical thinking patterns, leading to more self-critical ratings after treatment, despite no subjective worsening. This interpretation is consistent with qualitative reports of heightened insight. While these findings differ from positive self-compassion changes reported in previous AVATAR feasibility work [14], both studies are underpowered, and these discrepancies underscore the need for larger, controlled trials. Overall, the intervention appears to target core maintaining mechanisms of eating disorders, including identity fusion with the eating disorder and affect regulation difficulties.

### Limitations

The small sample size is typical of feasibility studies [31,49], and while it enabled qualitative exploration, it limits generalizability and likely inflates effect sizes. Results on clinical outcomes should be interpreted cautiously, given that participants continued to receive standard treatment alongside the VR intervention; therefore, any observed changes cannot be definitively attributed to the VR intervention. A further limitation was missing data caused by REDCap entry errors, resulting in incomplete IDEA and EDQLS scores. Additionally, small sample sizes for certain VAAS subscales limited the interpretation of the action subscale. Only 1 participant had a diagnosis of bulimia nervosa, and no male participants took part in the study, limiting the generalizability of acceptability and feasibility findings to a broader population with eating disorders; the results primarily reflect female individuals with anorexia nervosa.

### Conclusions

This study provides early evidence that VR-based avatar therapy is a feasible and acceptable approach as an add-on to treatment as usual for patients with long-standing eating disorders, complementing traditional cognitive and imagery-based methods [50,51] by virtually externalizing the eating disorder voice and facilitating direct, immersive confrontation. Potentially, immersive VR delivery may enhance realism and emotional engagement. The intervention may be particularly valuable for patients with entrenched symptomatology, for whom existing therapies yield limited gains.

On the basis of these findings, a full-scale randomized controlled trial is underway to evaluate efficacy, with additional attention to optimizing safety protocols, session structure, and technical reliability [52]. Future work should also investigate individual predictors of tolerance to exposure intensity and explore the potential for adaptive pacing.

## Data Availability

The datasets generated or analyzed during this study are not publicly available due to the small sample size and the resulting risk of participant re-identification but are available from the corresponding author on reasonable request.

## Appendix

Multimedia Appendix 1 CONSORT checklist extension for pilot and feasibility trials.

Multimedia Appendix 2 TIDieR checklist.

Multimedia Appendix 3 SRQR checklist.

Multimedia Appendix 4 Exploratory outcome findings.

## Abbreviations

CONSORT: Consolidated Standards of Reporting Trials
CSQ: Client Satisfaction Questionnaire
EDE-Q: Eating Disorder Examination Questionnaire
EDQLS: Eating Disorder Quality of Life Scale
IDEA: Identity and Eating Disorder Questionnaire
REDCap: Research Electronic Data Capture
SCS: Self-Compassion Scale
SRQR: Standards for Reporting Qualitative Research
TIDieR: Template for Intervention Description and Replication
VAAS: Voices Acceptance and Action Scale
VR: virtual reality
