# Supplementary material for: Virtual Reality–Based Avatar Intervention for Eating Disorders: Mixed Methods Feasibility Study
Source: JMIR Form Res. 2026 Mar 24;10:e88445. doi: 10.2196/88445 (PMC13058530; doi:10.2196/88445)
Supplement: Multimedia Appendix 4 [file formative_v10i1e88445_app4.pdf]

Multimedia appendix 4. Exploratory outcome findings from a single-arm feasibility study of virtual reality-based avatar therapy for eating disorders conducted at the Mental Health Centre Copenhagen, Copenhagen University Hospital, Denmark, between June 2023 and January 2024 (N=10).

**Outcomes**	**Participants, n/N (%)**	**Baseline measurements**		**Post-treatment measurements**		**Change (post-Treatment measurements - baseline measurements)**		***P*-value 2-sided**	**Hedges *g***
		Mean (SD)	Median (IQR)	Mean (SD)	Median (IQR)	Mean (SD)	95% CI		95% CI
**Eating Disorder Examination Questionnaire**	9/10 (90)	3.37 (1.64)	3.98 (2.98-4.13)	2.66 (1.59)	2.89 (2.51-3.46)	−0.71 (0.65)	−1.21 to −0.21	.01	−0.99 (−1.74 −0.24)
**Psychotic Symptoms Rating Scales**	9/10 (90)	24.78 (4.97)	24.50 (23.25-29.00)	20.33 (8.72)	25.00 (17.00-26.00)	−4.44 (8.14)	−10.70 to −1.82	.14	−0.49 (-1.13 to 0.14)
**Body Shape Questionnaire**	9/10 (90)	114.33 (42.76)	127.00 (91.00-133.00)	102.55 (41.10)	110.00 (93.00-120.00)	−11.78 (16.66)	24.58 to 1.04	.07	−0.64 (−1.30 to 0.02)
**Eating Disorder Quality of Life Scale**	9/10 (90)	80.55 (18.45)	79.00 (69.75-92.00)	78.00 (34.35)	85.00 (59.00-97.00)	−2.55 (24.46)	21.35 to 6.25	.76	−0.09 (−0.67 to 0.50)
**Self-Compassion Scale**									
Self-kindness	9/10 (90)	2.26 (0.62)	2.4 (2.05-2.40)	2.44 (0.90)	2.4 (2.0-3.2)	0.17 (0.51)	−0.22 to 0.56	.33	0.31 (−0.30 to 0.26)
Self-judgment	9/10 (90)	4.22 (0.57)	4.3 (3.70-4.55)	2.13 (0.70)	2.2 (2.00-2.40)	−2.11 (1.14)	−2.99 to −1.23	< .001	−1.66 (−2.63 to 0.69)
Common humanity	9/10 (90)	2.97 (0.82)	3.13 (3.00-3.50)	3.25 (0.96)	3.50 (2.75-4.00)	0.27 (0.52)	−0.13 to 0.67	.14	0.48 (−0.15 to 1.11)

Isolation	9/10 (90)	3.91 (0.67)	3.88 (3.31-4.25)	2.66 (0.87)	2.75 (2.50-3.25)	−1.25 (1.44)	−2.36 to −0.14	.03	−0.78 (−1.47 to 0.09)
Mindful-ness	9/10 (90)	2.94 (0.88)	3.00 (2.31-3.56)	3.13 (1.01)	3.25 (2.50-3.75)	0.19 (0.37)	−0.09 to 0.47	.15	0.47 (−0.16 to 1.10)
Overidenti-fication	9/10 (90)	4.00 (0.47)	4.00 (3.81-4.19)	2.33 (0.48)	2.5 (1.75-2.75)	−1.66 (0.86)	2.32 to −1.00	< .001	−1.74 (−2.74 to −0.74)
Total score	9/10 (90)	3.39 (0.31)	3.46 (3.14-3.60)	2.66 (0.70)	2.82 (2.14-3.15)	−0.73 (0.58)	1.18 to −0.28	.005	−1.14 (1.93 - 0.35)
**Beliefs About Voices Questionnaire Revised**									
Omni-potence	7/10 (70)	12.00 (2.31)	11.50 (11.00-13.75)	8.85 (5.18)	10 (6.50-12.00)	−3.14 (4.77)	7.55 to 1.27	.13	−0.57 (−1.28 to 0.14)
Malevolent	7/10 (70)	11.85 (3.44)	14 (10.00-14.75)	12.14 (2.12)	12 (11.50-12.50)	0.28 (2.56)	−2.09 to 2.65	.77	0.10 (−0.55 to 0.74)
Engage-ment affective	7/10 (70)	5.57 (3.10)	5.5 (4.25-7.50)	3.14 (2.61)	3 (1.00-5.50)	−2.42 (2.14)	−4.40 to −0.44	.02	−0.98 (−1.80 to −0.16)
Benevolent	7/10 (70)	5.42 (2.94)	4.50 (4.00-6.50)	4.14 (2.54)	4.00 (3.00-6.00)	−1.28 (3.81)	−0.89 to 3.17	.40	−0.29 (−0.96 to 0.37)
Engage-ment behavioral	7/10 (70)	2.42 (1.27)	2.00 (1.25-3.75)	1.28 (1.50)	1.00 (0.00-2.00)	−1.14 (2.19)	−3.17 to 0.89	.21	−0.45 (−1.14 to 0.23)
Resistance affective	7/10 (70)	6.58 (2.76)	6.5 (5.25-7.00)	7.00 (2.89)	8.00 (4.00-9.50)	0.42 (2.76)	−2.13 to 2.97	.69	0.14 (−0.51 to 0.78)
Resistance behavioral	7/10 (70)	8.71 (4.15)	7.00 (5.25-12.25)	11.00 (3.37)	10.00 (10.00-13.50)	2.29 (4.38)	−1.77 to 6.33	.21	0.45 (−0.23 to 1.14)
**Voice Power Differential Scale**	7/10 (70)	22.14 (5.21)	23.00 (17.75-25.75)	17.71 (5.28)	18.00 (15.00-19.00)	−4.42 (2.14)	6.40 to −2.44	.002	−1.63 (−2.59 to −0.67)

**Voice Acceptance and Action Scale (Section A)**	7/10 (70)	26.28 (4.61)	27.50 (22.25-28.75)	28.57 (4.76)	27.00 (25.50-32.00)	2.28 (3.30)	0.77 to 5.33	.11	0.60 (−0.12 to 1.32)
**Hospital Anxiety and Depression Scale**									
Anxiety	7/10 (70)	13.57 (3.20)	13.00 (9.50-16.25)	11.85 (3.53)	14.00 (10.50-14.00)	−1.71 (3.72)	5.15 to 1.73	.27	0.40 (−1.07 to 0.28)
Depression	7/10 (70)	6.85 (3.33)	7.50 (6.25-11.50)	5.57 (2.99)	6.00 (3.50-8.00)	−1.28 (2.69)	3.77 to 1.21	.25	−0.42 (−1.10 to 0.27)
**General Self-Efficacy Scale**	7/10 (70)	22.71 (5.50)		23.71 (6.52)		1.00 (2.88)	1.66 to 3.66	.40	0.30 (−0.36 to 0.97)
**Emotion Regulation Questionnaire**									
Cognitive reappraisal	7/10 (70)	3.90 (1.38)	4.08 (2.67-4.50)	4.88 (0.83)	4.50 (4.33-5.42)	0.97 (0.79)	0.24 to 1.70	.04	0.87 (0.08 to 1.66)
Expression suppression	7/10 (70)	3.78 (1.44)	4.25 (2.81-5.00)	3.00 (1.41)	3.00 (2.13-3.75)	−0.78 (1.14)	1.83 to 0.27	.12	0.60 (−1.31 to 0.11)
**Identity and Eating Disorder Questionnaire**	7/10 (70)	2.14 (0.63)	2.07 (1.92-2.52)	1.80 (0.49)	1.81 (1.50-2.11)	−0.34 (0.42)	0.73 to 0.05	.08	0.71 (−1.45 to 0.04)
**Behavior Rating Inventory of Executive Function**	7/10 (70)	44.42 (26.22)	46.00 (33.50-66.25)	47.57 (20.60)	45.00 (40.00-56.00)	3.15 (10.43)	6.50 to 12.80	.46	0.26 (−0.40 to 0.92)
